# Study on the technology of brewing red raspberry wine by using new immobilized yeast technology

**DOI:** 10.1038/s41598-022-25410-z

**Published:** 2022-12-09

**Authors:** Miao Liu, Xiaoyu Qin, Xiaotong Wu

**Affiliations:** School of Life Sciences, Inner Mongolia University, Hohhot, Inner Mongolia China

**Keywords:** Microbiology, Applied microbiology, Biological techniques, Microbiology techniques

## Abstract

The aim of the present study was to establish a new process for brewing red raspberry wine with immobilized yeast and improve the value of red raspberry. Using the spore filaments of *P. chrysogenum* as a carrier to co-culture with yeast to obtain immobilized yeast. And the preparation method of immobilized yeast was optimized by single-factor experiment and orthogonal experiments. And the red raspberry wine was brewed in these conditions. The result shows that the optimal preparation conditions for the immobilized yeast of *P. chrysogenum* were 5 g/L gluconic acid, 5 g/L yeast extract, and the addition amount of *P. chrysogenum* spores 1 × 10^5^ cfu/ml. Compared with free yeast, the immobilized yeast of *P. chrysogenum* has better fermenting ability, can better keep the anthocyanins and polyphenols and other active ingredients of red raspberry. The quality of the wine was improved due to increase in aroma components such as alcohols and esters. The immobilized yeast of *P. chrysogenum* maintained good fermentation performance after three consecutive fermentations.

## Introduction

Red raspberry (*Rubus idaeus *L.) is an aggregated berry of the genus Rubus of the family Rosaceae, also known as "raspberry", "Marin" and so on. They are widely distributed in Europe and likes a temperate climate^1^. Red raspberries are rich in polysaccharides, anthocyanins, ellagic acid, raspberry ketones and other substances^2^, which have antibacterial, antioxidant, anti-inflammatory, anticancer and other effects^3–5^, and have highly nutritive value and great value of health protection. Red raspberries are not easy to preserve due to soft fruit and easy breeding of botrytis. Their shelf life is only 2–3 days, and they are also easily spoiled during transportation^6^. Red raspberry wine brewing can prolong their shelf life and improve product value of red raspberry. At present, the fermentation technology of red raspberry wine is still earlier. At present, the fermentation process of red raspberry wine is still earlier and needs to be further developed.

Immobilized yeast technology refers to the use of suitable materials, physical or chemical methods to reserve live free yeast cells in a certain space, and to achieve multiple recycling and multiple continuous fermentation techniques^7^. It is a kind of immobilized cell technology. The immobilized cell technology has been widely concerned since the end of the nineteenth century. After more than 20 years of research and development history, the immobilized yeast technology has also been developed by leaps and bounds. According to different needs, different yeast immobilization materials and methods can be used^8^. Compared with free yeast, immobilized yeast has the advantages of high fermentation activity and repeatable fermentation. However, the traditional immobilized yeast has some shortcomings in the brewing process of red raspberry wine, such as unstable immobilized material and easy influence on wine quality.

As a new type of yeast immobilization technology, yeast biocapsule technology can realize co-immobilization between yeast and filamentous fungi, and yeast cells are immobilized on the inactivated filamentous fungi mycelium by attachment. Both *Rhizopus* and *Penicillium* mycelia can be used to prepare yeast biocapsules^9,10^. The inactivated filamentous mycelium, as an immobilized carrier, is chemically inert, and has less impact on the quality of fruit wine than other organic carriers, and the loose porous structure has good heat and mass transfer properties, which has a great impact on the physiological and metabolic properties of yeast cells. The influence of fermented wine is also small, so it is more conducive to ensure the quality and flavor formation during the fermentation process of wine^11^.

The aim of this paper is to optimize the immobilization method of *P. chrysogenum* encapsulated yeast, use the optimized immobilized yeast to ferment red raspberry wine, and explore the effect of the immobilization system on the fermentation process, flavor and quality of red raspberry wine. To provide theoretical basis for using immobilized yeast to ferment red raspberry wine.

## Materials and methods

### Materials and main reagents

Red raspberries: the variety is Haritiz, purchased from the vegetable base in Shebiya Village, Jinhe Town, Saihan District, Hohhot City, Inner Mongolia.

*Penicillium chrysogenum: BNCC 336,234, Beina Chuanglian Biotechnology Co., Ltd.*Active Dry Active dry yeast 71B: Saccharomyces cerevisiae, E491, Laman, France.

All the experiments were performed in accordance with relevant guidelines and regulations.

The main chemical reagents used are shown in Table 1.

**Table 1:** Experimental reagents.

Chemical reagent	Level	Factory
Glucose	Analytically pure	Tianjin Zhiyuan Chemical Reagent Co., Ltd
Lactose	Analytically pure	Tianjin Fengchuan Chemical Reagent Technology Co., Ltd
Sucrose	Analytically pure	Tianjin Damao Chemical Reagent Factory
Glycerin	Analytically pure	Tianjin Beilian Fine Chemicals Development Co., Ltd
Peptone	Biochemical reagents	Beijing Auboxing Biotechnology Co., Ltd
Gluconic acid	Food grade	Zhengzhou Yuhe Food Additives Co., Ltd
Yeast extract	Analytically pure	Oxoid UK
Urea	Analytically pure	Tianjin Fengchuan Chemical Reagent Technology Co., Ltd
Ammonium sulfate	Analytically pure	Tianjin Guangfu Technology Development Co., Ltd
Potassium nitrate	Analytically pure	Sinopharm Group Chemical Reagent Co., Ltd
Calcium chloride	Analytically pure	Tianjin Fengchuan Chemical Reagent Technology Co., Ltd
Sodium chloride	Analytically pure	Tianjin Fengchuan Chemical Reagent Technology Co., Ltd
Potassium chloride	Analytically pure	Tianjin Fengchuan Chemical Reagent Technology Co., Ltd
Potassium dihydrogen phosphate	Analytical grade	Tianjin Yongda Chemical Reagent Co., Ltd
Dipotassium hydrogen phosphate	Analytical grade	Tianjin Damao Chemical Reagent Factory
Magnesium sulfate	Analytical grade	Tianjin Zhiyuan Chemical Reagent Co., Ltd
Chloramphenicol	U.S. Pharmacopeia Grade	Beijing Coolbo Technology Co., Ltd

### Main instruments and equipment

The main equipment used is shown in Table 2.

**Table 2:** Experimental equipments.

Equipment	Model	Factory
Dual Incubation Shaker	BS-2E	Changzhou Langyue Instrument Manufacturing Co., Ltd
Biochemical incubator	SHP-250	Shanghai Pein Experimental Instrument Co., Ltd
Eppendorf centrifuge	5804	Ebende China Co., Ltd
Clean workbench	DL-CJ-2N	Beijing Donglian Haer Instrument Manufacturing Co., Ltd
pH meter	PHS-3C	Shanghai INESA Scientific Instrument Co., Ltd
Texture analyzer	TMS-Pilot	US FTC Corporation
Electrothermal constant temperature oscillating water tank	DKZ-1	Shanghai Yiheng Technology Co., Ltd
Ultrasonic cleaner	PS-20	Shenzhen Jiekang Ultrasonic Cleaning Equipment Co., Ltd

### Solution preparation

Ringer's solution: 8.6 g sodium chloride, 0.3 g potassium chloride, 0.28 g calcium chloride, 1L distilled water.

Neutral potassium phosphate buffer: 7.02 g dipotassium hydrogen phosphate, 2.62 g potassium dihydrogen phosphate, 1L distilled water.

Preparation of 25 mg/mL chloramphenicol solution: Dissolve 250 mg chloramphenicol in absolute ethanol, dilute to 10 mL, divide into small portions and store in − 20 °C refrigerator.

Immobilized yeast medium: 5 g/L gluconic acid, 5 g/L yeast extract, 0.5 g/L magnesium sulfate, 25 mg/L chloramphenicol, dissolved in neutral potassium phosphate buffer.

### *P. chrysogenum* and immobilized yeast culture methods

Cultivation of P. chrysogenum: The activated *Penicillium chrysogenum* was inoculated on PDA plate medium by three-point method, and cultured at 25 °C for 7 d^12^.

Cultivation of Immobilized Yeast: Saccharomyces cerevisiae 71B added at 4 × 10 cfu/mL, *P. chrysogenum* spores at 6 × 10 cfu/mL, medium bottled at 40 mL/100 mL, and cultured in shake flasks at 150 rpm for 7 d.

### Preparation method of immobilized yeast

Add an appropriate amount of sterile water to the PDA medium of *Penicillium chrysogenum* grown for 7 days, gently scrape the surface of *Penicillium chrysogenum* filaments with a spatula, pour the mixture into a sterile conical flask, and repeat the operation for 3 Second-rate. Shake the collected mycelial spore mixture, filter the mycelium with sterile gauze, and the obtained filtrate is the spore suspension of *Penicillium chrysogenum.* Yeast was added for co-cultivation, and the yeast was encapsulated by *Penicillium chrysogenum* to produce immobilized yeast. The immobilized yeast needs to be inactivated by *Penicillium chrysogenum* before use^12^.

### Optimization of preparation conditions for immobilized yeast

Carbon source optimization: keep the basic immobilized yeast medium and culture method unchanged, use 3 g/L ammonium sulfate and 2 g/L yeast extract as nitrogen sources, and use glucose, sucrose, lactose, gluconic acid and glycerol as carbon sources respectively. The addition amount is 5 g/L.

Nitrogen source optimization: other conditions remain unchanged, 5 g/L gluconic acid is used as carbon source, 2 g/L yeast extract is added, urea, ammonium sulfate, potassium nitrate, yeast extract and peptone are used as nitrogen sources, and the amount added is 3 g/L.

Optimization of *P. chrysogenum* spore addition: 5 g/L gluconic acid was used as carbon source, 5 g/L yeast extract was used as nitrogen source, and *P. chrysogenum* spore addition was 6 × 10^4^/mL and 1 × 10^5^/mL, respectively., 6 × 10^5^/mL, 1 × 10^6^/mL, 6 × 10^6^/mL.

### Physical property detection of immobilized yeast

Determination of the diameter of immobilized yeast: 10 mycelial balls were randomly selected and arranged in a straight line. Under the premise of maintaining the shape of the mycelial ball, an appropriate amount of filter paper was used to absorb the excess water on the surface of the mycelial ball. Measure the treated mycelium ball with a vernier caliper, and read the diameter value^11^.

Determination of the mechanical strength of immobilized yeast (texture analyzer method): The determination was carried out in the TPA mode of the texture analyzer, and the instrument probe was a universal cylindrical probe (diameter 45 mm; length 40 mm). The parameters of the TPA test program are set as the test speed of 100 mm/min, the deformation amount of 50%, the volume sensing element range of 25 N, the initial force of 0.04 N, and the pause time of 2 s. The measurement parameters are hardness and elasticity. Place the randomly selected immobilized yeast on the test bench. On the premise of maintaining the shape of the mycelial ball, use filter paper to absorb the excess water on the surface of the mycelial ball. After each test, wipe the probe clean before proceeding to the next test. Each test sample was measured 3 times, and the results were averaged.

Determination of the number of yeast encapsulated by immobilized yeast: 1 g of immobilized yeast was ground and crushed in a mortar, mixed with 10 mL of sterilized Ringer's solution, and shaken at 100 rpm for 30 min on an electrothermal constant-temperature shaking water tank. Sonicate in an ultrasonic apparatus for 1 min. Dilute the mixture appropriately and count using a hemocytometer^12^.

Determination of Leakage Count of immobilized yeast: 1 g of immobilized yeast was mixed with 10 mL of sterilized Ringer's solution and shaken at 100 rpm for 30 min on an electrothermal constant temperature shaking water tank. The initial concentration of the mixture was counted using a hemocytometer^12^.

### Comparison of between immobilized yeast and free yeast

The optimal red raspberry wine brewing process is the temperature of 26 °C, the sugar content of 220 g/L, and the immobilized yeast inoculum of 1.2%. Under these conditions, immobilized yeast and free yeast were fermented respectively, and the raspberry wines brewed by different fermentation methods were compared and analyzed in terms of changes in residual sugar, alcohol content, aroma components, and sensory evaluation.

Afterwards, the immobilized yeast was fermented for three times, and the repeated fermentation performance of the immobilized yeast was evaluated from the changes of residual sugar and alcohol content.

### Detection of red raspberry wine

Alcohol test: The alcohol content test refers to GB5009.225-2016 "National Food Safety Standard Determination of Ethanol Concentration in Wine".

Aroma component detection: 1 g NaCl was placed in a 20 ml headspace vial, and the cap was tightened. After equilibrating at 60 °C for 5 min in stirring mode, headspace extraction was performed at 60 °C for 20 min with a solid-phase microextraction needle, and then desorbed at the injection port for 5 min. The chromatographic column is HP-INNOWAX capillary column (30 m × 0.25 mm × 0.25 μm); the carrier gas is He, the flow rate is 1 mL/min, the separation ratio is 5:1; the injection temperature is 250 °C; the heating program is the initial temperature of 40 °C, hold for 5 min, increase to 250 °C at 8 °C/min, hold for 5 min.Mass spectrometry conditions: EI ionization source, energy 70 eV; ion source temperature 230 °C, quadrupole temperature 150 °C, interface temperature 250 °C, scanning range 30–400 m/z.

### Charting

All charts in this article are drawn by origin, or drawn by the test instrument independently.

## Results and discussion

### Optimization of preparation conditions for immobilized yeast

#### Carbon source optimization

The type of carbon source added has an effect on the size of the immobilized yeast and the clarity of the medium (Table 3). The diameters of the immobilized yeast from large to small were lactose, glycerol, gluconic acid, glucose, and sucrose. At the same time, the larger the diameter, the smaller the number of immobilized yeast. The carbon source had no obvious effect on the surface state of the immobilized yeast, and the surface of the immobilized yeast with different carbon sources is smooth. However, different carbon sources had an effect on the clarity of immobilized yeast culture medium. When using glucose, sucrose, and glycerol, the medium is cloudy because the carbon source used is suitable for yeast growth^11^. Simultaneously yeast overgrowth causes the medium to be cloudy and inhibits the growth of *P. chrysogenum.* When lactose is used, the medium is yellow, while when gluconic acid is used, the medium is clear and normal. Therefore, considering the growth state of immobilized yeast, gluconic acid is determined as the best carbon source.

**Table 3:** Effect of different carbon sources on immobilized yeast.

Carbon source	Diameter (mm)	Immobilized yeast growth state
Glucose	1.597 ± 0.105	Cloudy, smooth, excessive
Sucrose	1.573 ± 0.112	Cloudy, smooth, excessive
Lactose	2.843 ± 0.237	Yellow, smooth, medium
Gluconic acid	1.958 ± 0.086	Clear, smooth, multiple
Glycerol	2.514 ± 0.160	Cloudy, smooth, medium

#### Optimization of nitrogen source

The type of nitrogen source added affects the size of the immobilized yeast and the surface state of the immobilized yeast (Table 4). The diameters from large to small are urea, peptone, yeast extract, (NH4)2SO4, KNO3. Consistent with the effect of adding different carbon sources on immobilized yeast, the larger the diameter, the less the number of immobilized yeast. The immobilized yeast surface was rough when urea and peptone were used, indicating that nitrogen sources could affect the surface state of immobilized yeast. Based on the diameter and surface state of immobilized yeast, yeast extract was determined as the best nitrogen source.

**Table 4:** Effect of different nitrogen sources on immobilized yeast.

Nitrogen source	Diameter (mm)	Immobilized yeast growth state
Urea	2.889 ± 0.053	Clear, rough, minority
(NH_4_)_2_SO_4_	1.892 ± 0.042	Clear, smooth, excessive
KNO_3_	1.723 ± 0.116	Clear, smooth, excessive
Yeast extract	2.225 ± 0.257	Clear, smooth, medium
Peptone	2.349 ± 0.121	Clear, rough, multiple

#### Optimum dosage of *Penicillium chrysogenum* spores

The mycelium of *P. chrysogenum*, as the carrier of immobilized yeast, has a direct impact on the preparation of immobilized yeast^12^. The size of the immobilized yeast is different due to the different amount of *P. chrysogenum* spores added are 6 × 10^4^ cfu/ml > 1 × 10^5^ cfu/ml > 6 × 10^5^ cfu /ml > 1 × 10^6^ cfu/ml > 6 × 10^6^ cfu/ml (Table 5). The addition amount of *P. chrysogenum* can affect the diameter and number of immobilized yeast^13^. In terms of the number of immobilized yeasts, the more *P. chrysogenum* spores are added, the more immobilized yeasts will be. When the amount of spores added is large, more spores can interact with the yeast in a unit volume and can interact quickly, so it is easier and faster to form more immobilized yeasts with small diameters. On the other hand, because the number of yeasts is basically constant, the more spores of *P. chrysogenum* are added, the less yeast can be effectively encapsulated by each immobilized yeast, and the volume is small. The medium with each concentration of *P. chrysogenum* spores was in a clear state and the surface was smooth, indicating that the addition of *P. chrysogenum* spores had no effect on the clarity of the medium, that is, the surface state of the immobilized yeast. Considering the quantity and size of immobilized yeast, the optimal spore addition range is 1 × 10^5^ cfu/ml ~ 6 × 10^5^ cfu/ml.

**Table 5:** Effect of the amount of spores of *P. chrysogenum* on immobilized yeast.

The amount of spores added (cfu_*_ml^−1^)	Diameter (mm)	Immobilized yeast growth state
6 × 10^4^	4.827 ± 0.163	Clear, smooth, minority
1 × 10^5^	3.932 ± 0.344	Clear, smooth, minority
6 × 10^5^	2.258 ± 0.240	Clear, smooth, medium
1 × 10^6^	1.912 ± 0.081	Clear, smooth, excessive
6 × 10^6^	1.129 ± 0.052	Clear, smooth, excessive

#### Orthogonal experiment

Based on the above, it is determined that the optimal carbon source is gluconic acid, the optimal nitrogen source is yeast extract, and the optimal addition amount of *P. chrysogenum* spores is 1 × 10^5^ cfu/ml ~ 6 × 10^5^ cfu/ml. Orthogonal optimization of the preparation conditions of immobilized yeast based on the optimal carbon source, nitrogen source and the addition amount of *P. chrysogenum* spores determined by single factor, design three-factor three-level orthogonal test of factors, the test level is shown in Table 6.

**Table 6:** Orthogonal test level.

Level	Factor		
	A Gluconic acid (g/L)	B Yeast extract (g/L)	C *P. chrysogenum* spore addition (cfu/ml)
1	2.5	2.5	1 × 10^5^
2	5	5	3.5 × 10^5^
3	7.5	7.5	6 × 10^5^

The factors affecting the number of encapsulated bacteria in immobilized yeast were B > A > C, and the factors affecting the number of leaking bacteria in immobilized yeast were C > A > B. According to the orthogonal results (Table 7), when the diameter is used as the selection standard, the optimal combination is A2B2C1, namely No. 5. When the number of encapsulated bacteria is used as the selection criterion, the optimal combination is A2B1C3, namely No. 4. When the number of leaking bacteria was used as the selection criterion, the optimal combination was A2B2C1, namely No. 5.

**Table 7:** Immobilized yeast optimized orthogonal test results.

Test	Factor			Diameter (mm)	Number of encapsulated bacteria (10^7^ cfu/g)	Leakage count (10^7^ cfu/g)
	A	B	C			
1	1	1	1	3.073	8.70	0.25
2	1	2	2	2.627	9.45	0.40
3	1	3	3	1.109	4.45	0.85
4	2	1	3	2.837	12.90	0.45
5	2	2	1	3.299	7.05	0.05
6	2	3	2	1.641	5.50	0.1
7	3	1	2	1.858	7.85	0.25
8	3	2	3	1.947	5.75	0.45
9	3	3	1	2.977	4.15	0.05
k_1_	2.270	2.589	3.116	–		
k_2_	2.592	2.624	2.042	–		
k_3_	2.261	1.909	1.964	–		
R_1_	0.331	0.659	1.152	–		
l_1_	7.533 × 10^7^	9.817 × 10^7^	6.633 × 10^7^			
l_2_	8.483 × 10^7^	7.417 × 10^7^	7.600 × 10^7^			
l_3_	5.917 × 10^7^	4.700 × 10^7^	7.700 × 10^7^			
R_2_	2.566 × 10^7^	5.117 × 10^7^	1.067 × 10^7^			
m_1_	0.500 × 10^7^	0.317 × 10^7^	0.117 × 10^7^			
m_2_	0.200 × 10^7^	0.300 × 10^7^	0.250 × 10^7^			
m_3_	0.250 × 10^7^	0.333 × 10^7^	0.583 × 10^7^			
R_3_	0.300 × 10^7^	0.033 × 10^7^	0.466 × 10^7^			

#### Effects of mechanical strength of immobilized yeast

**Figure 1:**
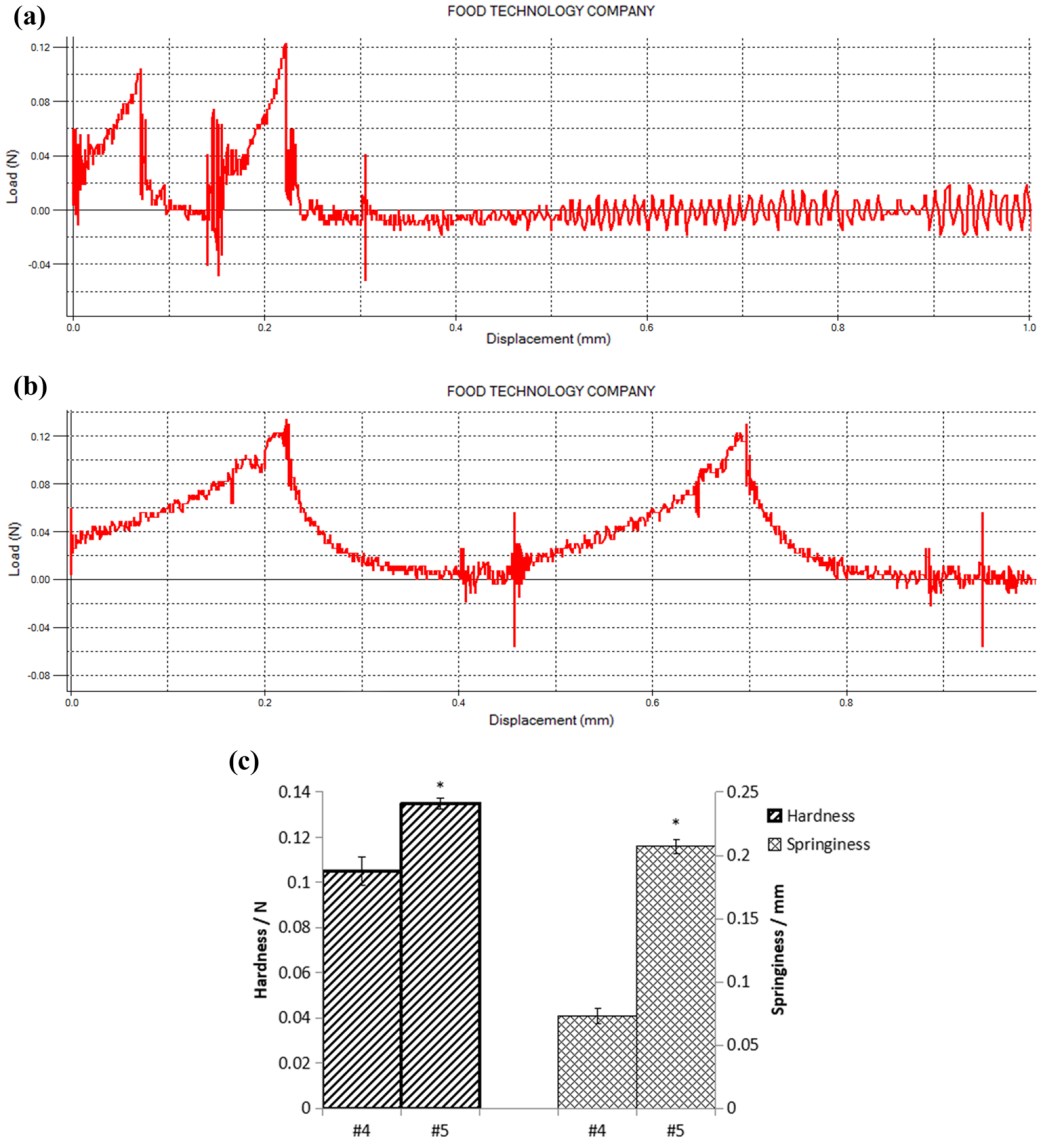
(**a**) Texture map of the 4th group of immobilized yeast. (**b**) Texture map of the 5th group of immobilized yeast. (**c**) Hardness and springiness of immobilized yeast.

Immobilized yeast will be subjected to different mechanical forces during the fermentation process, resulting in crushing damage or scraping damage^14^, so the mechanical strength of immobilized yeast has an important impact on the performance and repeatability of immobilized yeast. The two groups of optimal combinations obtained from the optimized orthogonal test of immobilized yeast, the test groups No. 4 and No. 5, were tested for mechanical strength (Fig. 1a,b). The comparison between the two was shown in Fig. 1c. It can be seen that the hardness of No. 4 is 0.105 N and the elasticity is 0.135 mm. The hardness of No. 5 is 1.29 times that of No. 4, and the elasticity is 1.5 times that of No. 4, which are 0.135 N and 0.207 mm, respectively.

Therefore, the optimal immobilized yeast was determined as the No. 5 test group, with 5 g/L of gluconic acid, 5 g/L of yeast extract, and 1 × 10^5^ cfu/ml of *P. chrysogenum* spores.

### Comparison between immobilized yeast and free yeast

#### Comparison of fermentation performance

**Figure 2:**
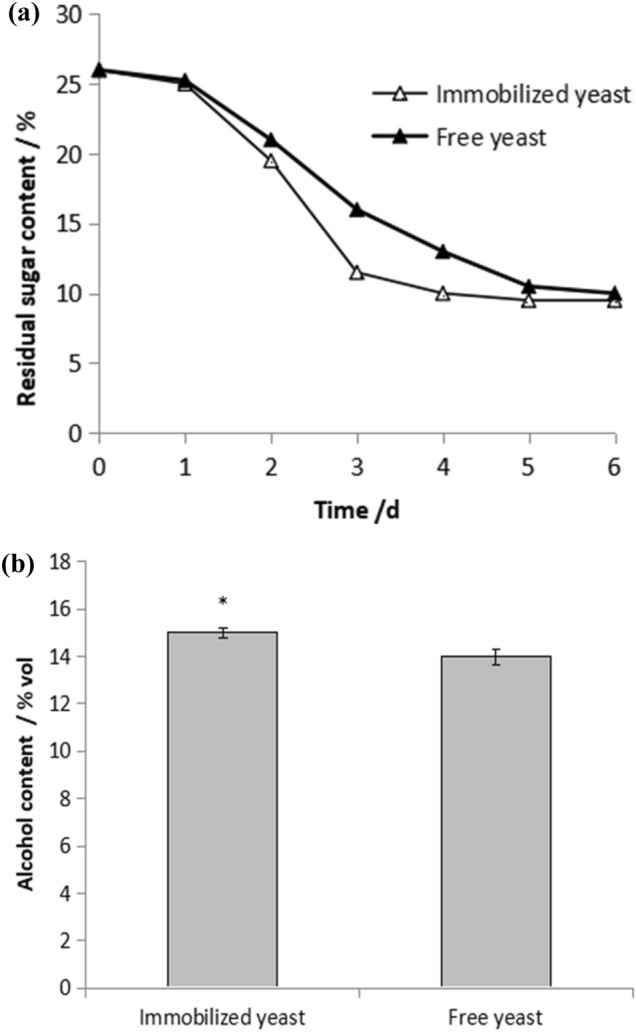
(**a**) Comparison of residual sugar content change between free yeast and immobilized yeast. (**b**) Comparison of alcohol content between free yeast and immobilized yeast.

The results of residual sugar and alcohol content of red raspberry wine brewed by immobilized yeast and free yeast are shown in Fig. 2a,b. It can be seen that the residual sugar content of free yeast is slightly higher than that of immobilized yeast, and the sugar consumption rate of immobilized yeast is higher than that of free yeast, indicating that the fermentation rate of immobilized yeast is higher than that of free yeast.

The alcohol content of red raspberry wine brewed by immobilized yeast is 15% vol, and it brewed by free yeast is 14% vol. It was also proved that with the same inoculum amount, the fermentation ability of immobilized yeast was better than that of free yeast.

#### Comparison of ingredients in brewing red raspberry wine

The total acid, anthocyanin, reducing sugar and polyphenol content of red raspberry wine brewed with immobilized yeast and free yeast were analyzed.

**Figure 3:**
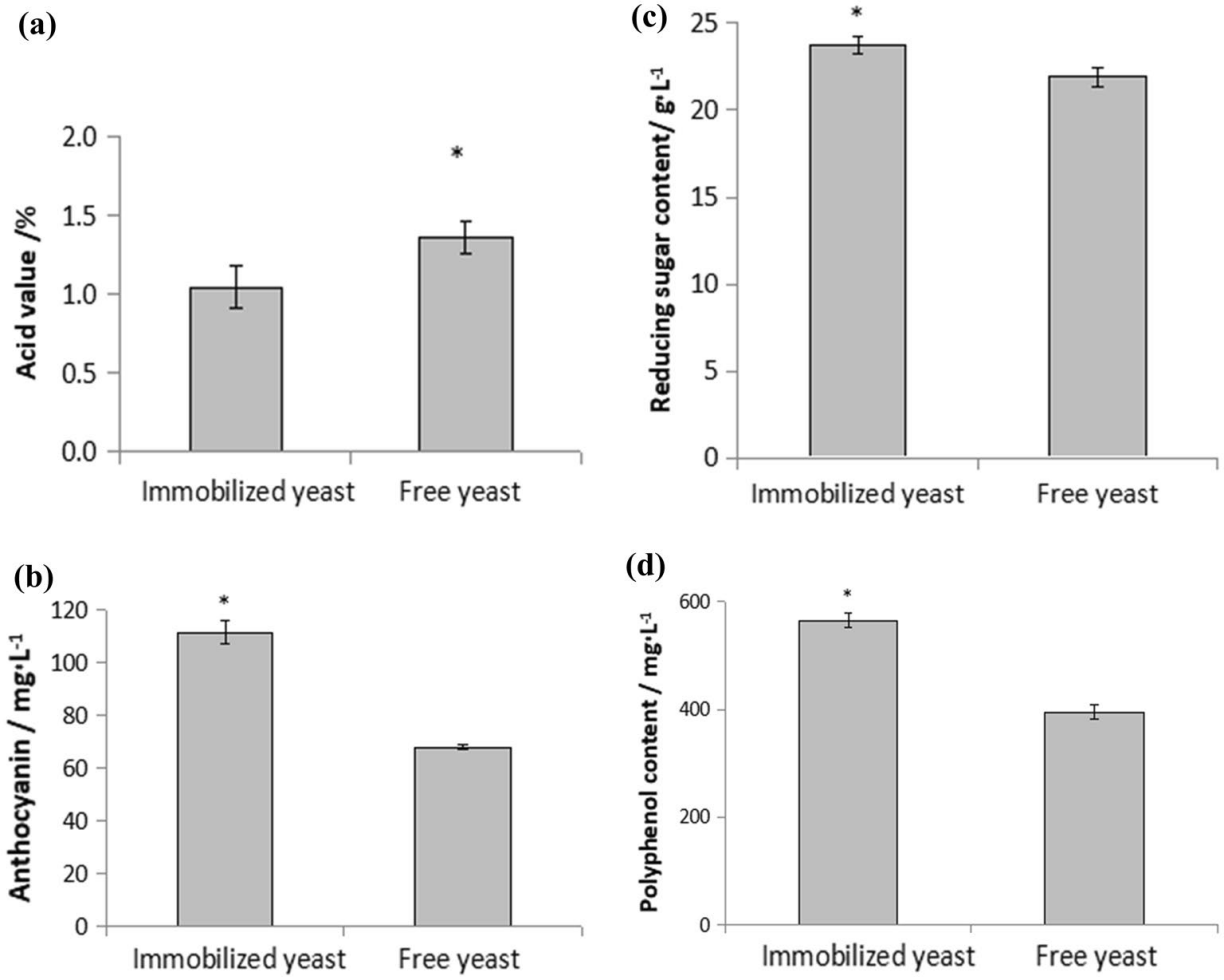
(**a**) Comparison of acid value between immobilized yeast and free yeast. (**b**) Comparison of anthocyanin content between immobilized yeast and free yeast. (**c**) Comparison of reducing sugar between free yeast and immobilized yeast. (**d**) Comparison of polyphenol content between free yeast and immobilized yeast.

It can be seen that the total acid content of the red raspberry wine brewed by immobilized yeast is 1.0438%, which is lower than 1.3687% of the red raspberry wine brewed by free yeast (Fig. 3a). That is, the acidity of red raspberry wine brewed by immobilized yeast is lower than that of free yeast, and the taste of red raspberry wine brewed by immobilized yeast is also better than that of free yeast.

The anthocyanin content of immobilized yeast brewed red raspberry wine was 111.604 mg/L, the reducing sugar content was 23.73 g/L, and the polyphenol content was 565.67 mg/L; the red raspberry wine brewed by free yeast was 68.020 mg /L, 21.93 g/L, 395 mg/L respectively (Fig. 3b,c,d).The reducing sugar content of red raspberry wine brewed by free yeast and immobilized yeast has little difference, but the content of anthocyanins and polyphenols in immobilized yeast is higher. It shows that immobilized yeast can better retain the active components of red raspberry wine compared to free yeast.

#### Comparison of aroma components in brewing red raspberry wine

**Figure 4:**
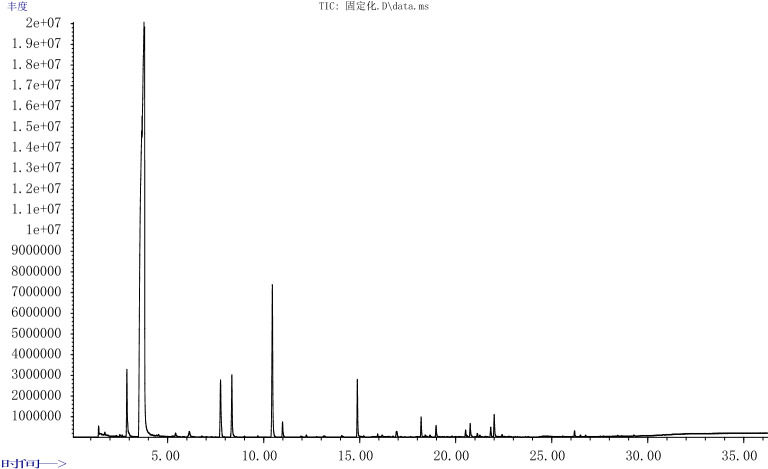
GC–MS total ion outflow Figure of aroma components of red raspberry wine fermented with Immobilized yeast.

**Figure 5:**
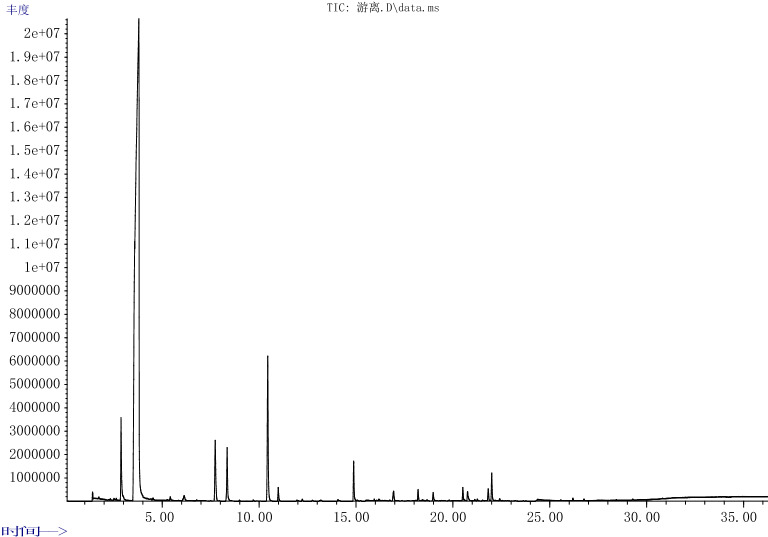
GC–MS total ion outflow Figure of aroma components of red raspberry wine fermented with free yeast.

Figures 4 and 5 for the GC–MS total ion efflux of aroma components in immobilized yeast and free yeast fermented red raspberry wine. A total of 40 main aroma components were detected in the two red raspberry wines, including 5 alcohols, 21 lipids, 3 acids, 3 alkenes, 3 aldehydes and ketones, 1 phenol, and 4 others. The two red raspberry wines have the same 22 kinds of aroma components, mainly alcohols and esters.

It can be seen from Table 8 that the main aroma components of the red raspberry wine brewed by immobilized yeast from high to low are isoamyl alcohol 6.8312%, isobutanol 2.8885%, ethyl acetate 2.7084%, isoamyl acetate 2.5946%, ethyl caprylate 2.0175%. The main aroma components of red raspberry wine brewed with free yeast from high to low are isoamyl alcohol 6.0499%, ethyl acetate 2.8018%, isobutanol 2.7780%, isoamyl acetate 2.0802%, ethyl caprylate 1.3425%.

**Table 8:** Main aroma components of red raspberry wine fermented with Immobilized yeast or free yeast.

	Name	Immobilized yeast content/%	Free yeast content/%
**Alcohols**			
1	Propanol	0.3891	0.3950
2	Isobutanol	2.8885	2.7780
3	Isoamyl alcohol	6.8312	6.0499
4	a-Ionol	0.3762	0.4533
5	Phenylethanol	0.8822	0.9710
**Esters**			
6	Ethyl acetate	2.7084	2.8018
7	Isobutyl acetate	0.1491	0.1330
8	Isoamyl acetate	2.5946	2.0802
9	Ethyl n-hexanoate	0.6160	0.5062
10	Ethyl octanoate	2.0175	1.3425
11	Ethyl sorbate	0.0702	0.0655
12	Ethyl caprate	0.6929	0.3870
13	Ethyl dodecanoate	0.1303	0.0499
14	Ethyl palmitate	0.2113	0.0864
15	Ethyl hex-4-enoate (other isomers)	0.0725	–
16	Octyl 4-ethylbenzoate	0.0435	–
17	Ethyl benzoate	0.0913	–
18	Cyclopropanecarboxylic acid, 2-phenylethyl ester	0.5557	–
19	E-11 hexadecenoic acid, Ethyl ester	0.0633	–
20	Ethyl glycolate	–	0.2444
21	Ethyl hexenoate	–	0.0649
22	Monoethyl malonate	–	0.4270
23	Phenethyl acetate	–	0.4501
24	Methyl caproate	–	0.0402
**Acids**			
25	Phthalic acid, Isobutyl ester nonyl	0.0405	–
26	5-Methylanthranilic acid	–	0.0370
27	Octanoic acid	–	0.0480
**Olefins, Alkanes**			
28	Diethoxydimethylsilane	0.0355	0.0467
29	Ethyl 9-decene	0.4748	0.3330
30	Dodecane	0.3573	–
**Aldehydes, Ketones**			
31	Acetaldehyde	0.1037	0.0471
32	α-ionone	0.0632	0.0614
33	β-ionone	0.0893	0.0832
**Benzene, Phenols**			
34	2,4-Di-tert-butylphenol	0.0404	0.0532
**Others**			
35	Diacetyl	0.0907	–
36	4-O-Methyl-α-D-Mannoside	0.2513	–
37	1-Acetylguanidine	–	0.0879
38	Bis(2,4-dimethylamino)pyrimidine	–	0.4954

**Figure 6:**
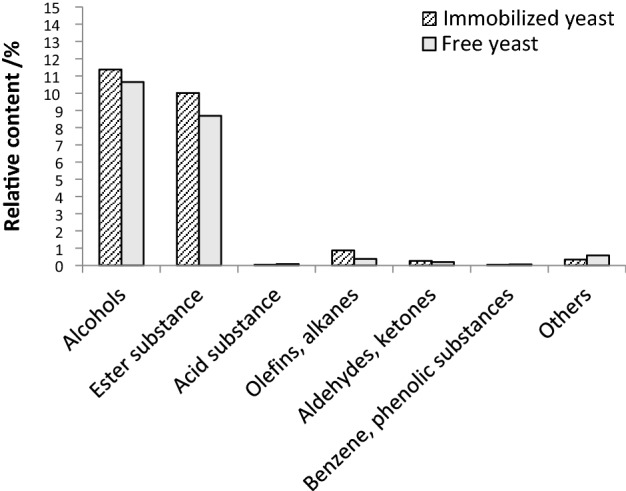
Aroma composition of red raspberry wine fermented with Immobilized yeast or free yeast.

The most abundant aroma component in both raspberry wines was isoamyl alcohol. The total alcohol content of red raspberry wine brewed by immobilized yeast was 11.3627%, which was higher than that of free yeast brewed red raspberry wine 10.6472%. The lipid content of the red raspberry wine brewed with immobilized yeast was 10.0166%, which was higher than that of the red raspberry wine brewed with free yeast, which was 8.6791%. Compared with free yeast fermentation (Fig. 6), immobilized yeast can significantly increase the ester content^15^. Other studies have also shown that the use of immobilized yeast for fermentation can promote the production of aroma compounds such as alcohols and esters^9^. In addition, the immobilized yeast also effectively reduced the content of volatile acids, which was beneficial to the quality maintenance of red raspberry wine^16^.

#### Continuous fermentation performance of immobilized yeast

**Figure 7:**
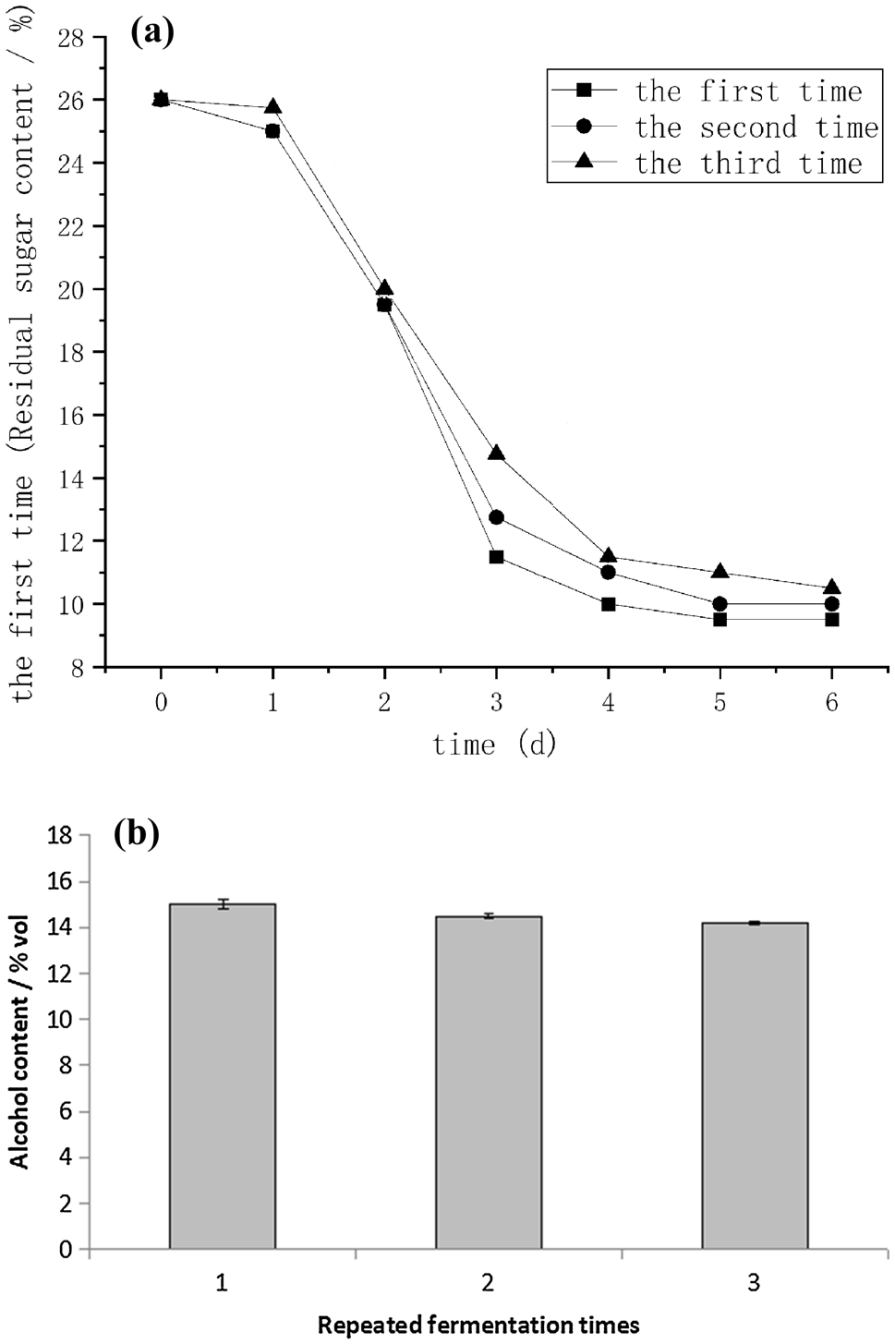
(**a**) Residual sugar change in continuous fermentation of immobilized yeast. (**b**) Alcohol change in continuous fermentation of immobilized yeast.

Figure 7a,b show the changes of residual sugar content and alcohol content of immobilized yeast for three consecutive fermentations. From the results, the residual sugar content of three consecutive fermentations was 9.5%, 10%, and 10.5%, respectively. Alcohol content the degrees were 15% vol, 14.5% vol, and 14.2% vol. With the increase of fermentation times, the residual sugar content increased slightly, and the alcohol content decreased slightly, but the changes were not obvious, indicating that the immobilized yeast of *P. chrysogenum* maintained good fermentation performance after three consecutive fermentations.

## Conclusion

The immobilized yeast of *P. chrysogenum* can be prepared by adding *P. chrysogenum* spores and co-culturing with yeast in a shaker flask. The optimal preparation conditions were determined by orthogonal optimization as 5 g/L of gluconic acid, 5 g/L of yeast extract, and 1 × 105 cfu/ml of *P. chrysogenum* spores. The obtained immobilized yeast has good physiological characteristics and mechanical properties.

In the fermentation process of red raspberry wine, the immobilized yeast of *P. chrysogenum* has better fermentation power than free yeast, and can better retain the active components such as anthocyanins and polyphenols in red raspberry wine, and at the same time obviously. The content of aroma components such as alcohols and lipids in red raspberry wine was improved. The immobilized yeast of *P. chrysogenum* maintained good fermentation performance after three consecutive fermentations.

## Data Availability

All data generated or analysed during this study are included in this published article.
